# Quantitative evaluation of meibomian gland dysfunction via deep learning-based infrared image segmentation

**DOI:** 10.3389/frai.2025.1642361

**Published:** 2025-10-29

**Authors:** Ziyang Yu, Zhijun Wei, Mini Han Wang, Jiazheng Cui, Jiaxiang Tan, Yang Xu

**Affiliations:** ^1^Beijing Institute of Technology, Zhuhai, China; ^2^School of Integrated Circuits and Electronics, Beijing Institute of Technology, Beijing, China; ^3^Zhuhai Institute of Advanced Technology Chinese Academy of Sciences (CAS), Zhuhai, China; ^4^Zhuhai People’s Hospital (The Affiliated Hospital of Beijing Institute of Technology, Zhuhai Clinical Medical College of Jinan University), Zhuhai, China; ^5^Department of Ophthalmology and Visual Sciences, Chinese University of Hong Kong, Hong Kong, Hong Kong SAR, China; ^6^Beijing Normal University—Hong Kong Baptist University United International College, Zhuhai, China

**Keywords:** image segmentation, meibomian gland dysfunction, dry eye disease, meibograpy, deep learning

## Abstract

In recent years, numerous advanced image segmentation algorithms have been employed in the analysis of meibomian glands (MG). However, their clinical utility remains limited due to insufficient integration with the diagnostic and grading processes of meibomian gland dysfunction (MGD). To bridge this gap, the present study leverages three state-of-the-art deep learning models—DeepLabV3+, U-Net, and U-Net++—to segment infrared MG images and extract quantitative features for MGD diagnosis and severity assessment. A comprehensive set of morphological (e.g., gland area, width, length, and distortion) and distributional (e.g., gland density, count, inter-gland distance, disorder degree, and loss ratio) indicators were derived from the segmentation outcomes. Spearman correlation analysis revealed significant positive associations between most indicators and MGD severity (correlation coefficients ranging from 0.26 to 0.58; *p* < 0.001), indicating their potential diagnostic value. Furthermore, Box plot analysis highlighted clear distribution differences in the majority of indicators across all grades, with medians shifting progressively, interquartile ranges widening, and an increase in outliers, reflecting morphological changes associated with disease progression. Logistic regression models trained on these quantitative features yielded area under the receiver operating characteristic curve (AUC) values of 0.89 ± 0.02, 0.76 ± 0.03, 0.85 ± 0.02, and 0.94 ± 0.01 for MGD grades 0, 1, 2, and 3, respectively. The models demonstrated strong classification performance, with micro-average and macro-average AUCs of 0.87 ± 0.02 and 0.86 ± 0.03, respectively. Model stability and generalizability were validated through 5-fold cross-validation. Collectively, these findings underscore the clinical relevance and robustness of deep learning-assisted quantitative analysis for the objective diagnosis and grading of MGD, offering a promising framework for automated medical image interpretation in ophthalmology.

## 1 Introduction

Meibomian gland dysfunction (MGD) is a common ophthalmic disease and one of the main causes of dry eye disease (DED). Its incidence rate can be as high as 50% globally, and it is particularly more prevalent among the female sex and older age (Stapleton et al., 2017). From a medical perspective, the pathological basis of MGD contains the complex interaction of structural changes in the gland, weakened secretion function, and inflammatory responses, leading to excessive tear evaporation and the aggravation of dry eye symptoms (Ban et al., 2013; Baudouin et al., 2016).

In order to achieve an accurate assessment to the severity of MGD, researchers and clinicians are increasingly relying on image analysis techniques such as infrared meibography and image segmentation to establish an objective and reproducible method (Tomlinson et al., 2011). Artificial intelligence (AI) technologies have demonstrated significant potential in the diagnosis of ophthalmic diseases. By integrating multi-source evidence, such as infrared MG imaging and clinical data, the accuracy and repeatability of MGD diagnosis have been significantly improved (Wang et al., 2024). AI applications based on mobile health platforms have promoted the early screening and dynamic monitoring of MGD (Wang et al., 2025b). Additionally, explainable AI has supported the automated diagnosis of ophthalmic diseases like MGD by optimizing model robustness (Wang et al., 2023a). Subjective symptom assessment and tear break-up time measurement as traditional medical diagnostic methods, are limited by the experience of clinicians and the temporary situation feedback, hardly to meet the requirements of precision medicine for objective and quantitative indicators (Wolffsohn et al., 2017). The early analysis and diagnosed methods of MG mainly relied on semi-automatic or manual segmentation (Koh et al., 2012), which had significant limitations. This method not only consumes a great deal of time but also leads to a waste of labor, and the segmentation effect was highly dependent on the image quality, making it challenging to meet the requirements of objective and reproducible diagnosis.

The rapid development of algorithmic technologies has brought new opportunities to this field, particularly the widespread application of deep learning in medical image segmentation. Deep learning models such as U-Net and DeepLab have demonstrated excellent performance in image segmentation tasks (Chen et al., 2018b; Ronneberger et al., 2015). By automatically identifying complex structures in images, they provide a feasible solution for the quantitative analysis of MG morphology and function (Lundervold and Lundervold, 2019). The analysis method of MG quantitative indicators based on image segmentation has overcome the subjectivity and inefficiency of manual operations, significantly improving the repeatability and consistency of MG assessment, and providing an objective basis for the early diagnosis and dynamic monitoring of MGD (Setu et al., 2021). The interdisciplinary innovation of this study is expected not only to enhance the efficiency of ophthalmic clinical diagnosis but also to provide technical references for automated analysis in other medical imaging fields.

The aim of this study is to develop a novel quantitative index extraction method based on segmentation results and explore its application value in the diagnosis of MGD. Specifically, DeepLabV3+, U-Net, and U-Net++ models will be used to process infrared MG images. Following the segmentation, a series of quantitative indicators, including innovative metrics such as gland area, density, width, distance between adjacent glands, degree of disorder, and loss ratio, as well as a novel principal component analysis-based approach for calculating gland length, width, and distortion to more accurately capture complex geometric features, will be calculated. The correlation of these indicators with MGD grade and their diagnostic efficacy will be systematically validated through Spearman correlation analysis, box plot visualization, and logistic regression models.

## 2 Related works

AI technologies have demonstrated remarkable potential in the field of ophthalmic disease diagnosis. In particular, significant breakthroughs have been achieved in the detection and grading of DED and ocular surface diseases. To comprehensively evaluate the function and morphology of MG, various clinical trials have been established. Currently, the assessment of gland secretion quality and expression is widely used as a key approach to evaluate MG function.

In clinical morphology, a study (Xiao et al., 2019) analyzed meibomian glands (MG) using infrared imaging and evaluated the correlation between their morphological characteristics including gland loss, length, thickness, density, and distortion, and the severity of MGD. The results showed that gland distortion and gland loss were highly sensitive indicators for MGD, with the areas under the curve (AUC) reaching 0.96 and 0.98, respectively. Another research (Lin et al., 2020) introduces a new method to measure the distortion of MG, defined as the ratio of the actual gland length to its straight-line length minus 1. The results demonstrated that MGD patients had significantly higher distortion values (*p* < 0.05), and when using the distortion of the middle eight glands as the criterion for diagnosing obstructive MGD, both sensitivity and specificity reached 100%. However, a major limitation of these two methods is their reliance on manual measurement, which may lead to potential errors and reduce efficiency.

Different from previous studies, some research has quantitatively analyzed the morphology and function of MG through infrared images, which is used for the diagnosis and grading of MGD (Llorens-Quintana et al., 2019; Deng et al., 2021). The algorithm proposed by Clara focuses on the upper eyelid and analyzes parameters such as length, width, and irregularity. It has been verified that this algorithm has lower variability and higher consistency compared with subjective assessment. Deng’s algorithm, on the other hand, analyzes the gland area ratio, diameter deformation index, tortuosity index, and signal index of the upper eyelid. When the combined parameters are used for diagnosing MGD, the AUC can reach 0.82, and the accuracy in grading is excellent. Both of these algorithms adopt image segmentation techniques, overcoming the limitations of subjective assessment and providing non-invasive and objective diagnostic tools for MGD, demonstrating the interdisciplinary potential of medical image analysis. However, relying on traditional image processing and segmentation techniques to analyze infrared images, both algorithms face limitations in segmentation accuracy when dealing with complex or irregular gland structures. Moreover, neither of them has overcome the deficiencies in analyzing the lower eyelid.

Furthermore, more and more scholars are using deep learning to analyze infrared MG images for the assessment of MGD, demonstrating the potential for automatic segmentation and morphological assessment. A pre-trained U-Net model was employed (Setu et al., 2021) to process medical images, achieving a Dice coefficient of 84%. This approach quantified features such as the number, length, width, and tortuosity of glands in both upper and lower eyelids. A Conditional Generative Adversarial Network based model was utilized (Khan et al., 2021) for gland segmentation. Through adversarial learning between the generator and discriminator, the model generated a confidence map, achieving a Jaccard index of 0.664, an F1 score of 0.825, and high correlation with manual analysis results. Two AI techniques semantic segmentation and object detection were applied (Swiderska et al., 2023) to quantify MG features, including length, area, and curvature. TransUnet combined with data augmentation was proposed (Lai et al., 2024) to enhance meibomian gland imaging analysis. By automatically calculating the proportion of white pixels in the MG and conjunctiva regions, an automatic meiboscore was achieved, which highly agreed with the judgment of professional physicians. Recent studies have enhanced the intelligence level of MGD diagnosis through contrastive learning augmented by knowledge graphs, integrating clinical feature cues (Han Wang et al., 2025). Prompt engineering has optimized the ability of AI models to recognize complex gland structures by designing clinically oriented prompts (Wang et al., 2025a). Additionally, explainable AI has provided reliable support for automated MGD detection by improving data quality and model transparency (Wang et al., 2023b).

## 3 Materials and methods

### 3.1 Materials

**Figure 1:**
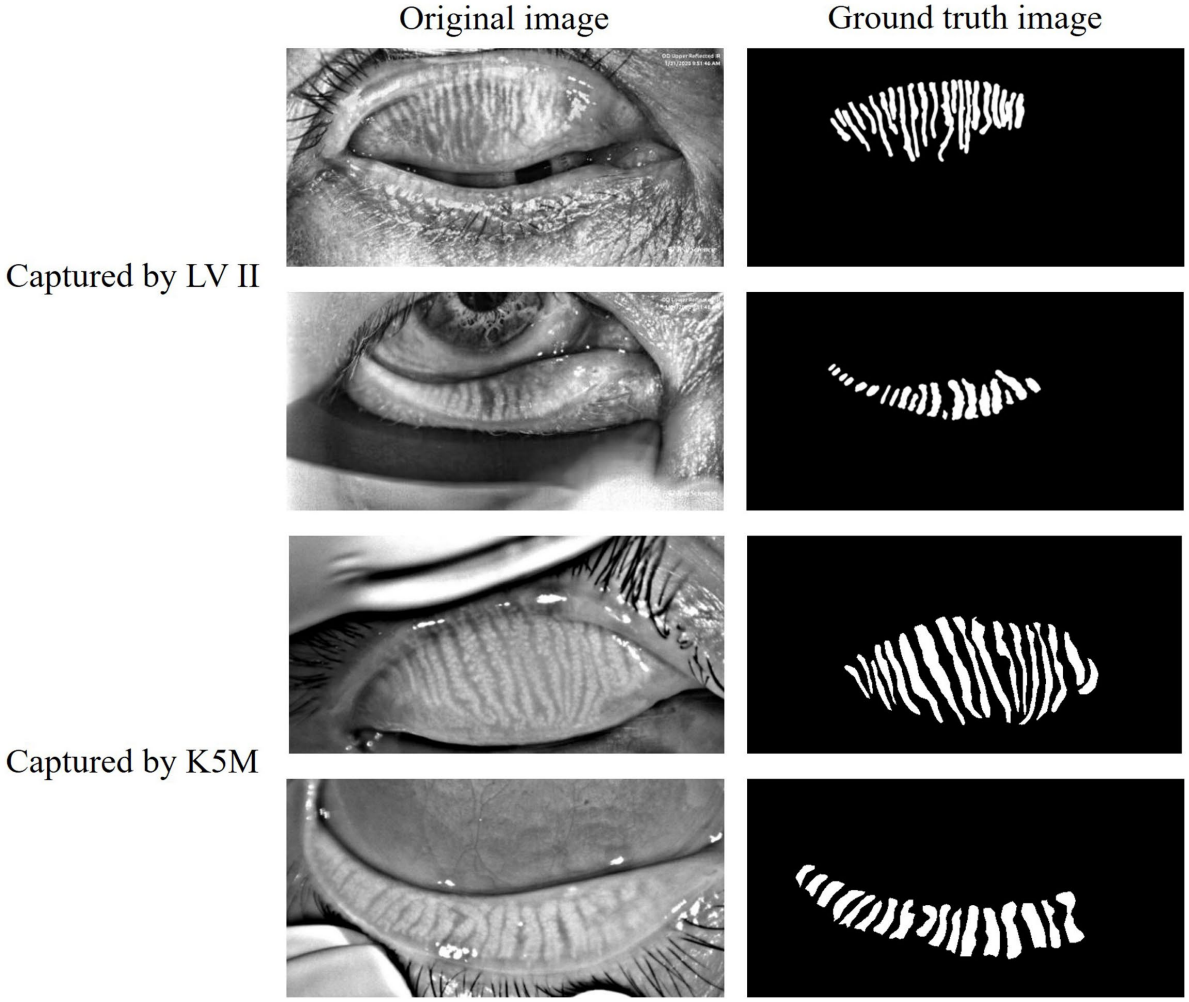
Case of original infrared meibography and their corresponding annotated images.

The data of this study on MGD analysis collected from multiple sources. The public data MGD-1 K (Saha et al., 2022) contains 1,000 infrared images of MG captured by the Lipi View II Ocular Surface Interferometer (LV II). The in-house data consists of a total of 265 anonymous clinical infrared MG images of eyelids, which were randomly collected from the database of the Oculus Keratograph 5 M (K5M; Oculus GmbH, Wetzlar, Germany) at Zhuhai People’s Hospital (The Affiliated Hospital of Beijing Institute of Technology, Zhuhai Clinical Medical College of Jinan University). These datasets were annotated for glands, eyelids, and MGD grade under the direct supervision of three MGD experts and specialized ophthalmologists. As shown in Figure 1, both the original infrared MG images and their corresponding annotated glands images are presented, clearly demonstrating the annotation quality of the datasets and the structural details of the MG. The grading of MGD adopts the criteria recommended by TFOS DEWS II (Craig et al., 2017), and comprehensive grading evaluation is carried out based on the severity of MGD and the morphological characteristics of MG. Specifically, the grading system evaluates gland morphology including architectural shape, structural variations such as tortuosity and curvature patterns, and gland loss severity which directly reflects MGD progression. Quantification of gland loss ratio serves as the fundamental morphological metric for assessment.

From a combined collection of 1,265 fully annotated infrared MG images derived from two datasets, we proportionally allocated 300 images to constitute the test set based on the original datasets ratio, which will be utilized for model performance evaluation and MGD grading. The remaining 965 images were partitioned into training and validation sets at an 8:2 ratio while maintaining the source data distribution, designated for deep learning model training and validation respectively, thereby ensuring all data partitions preserve equilibrium in original data representation. Because of the inconsistent sizes of the images in the data, all images are uniformly resized to 1,280 × 640 pixels, with no rotation, flipping, or other image preprocessing methods applied to preserve original information, reduce information loss, and lower computational costs. All experiments were conducted using PyTorch 2.6.0 and Python 3.12.9 on a computing platform equipped with four NVIDIA A100-PCIe 40GB GPUs running Ubuntu 20.04.

### 3.2 Model architecture

In this study, DeepLabV3+, U-Net, and U-Net++ were selected as image segmentation models due to their established efficacy and complementary strengths in medical image segmentation, particularly for processing complex anatomical structures like MG. It should be noted that the exclusion of other advanced medical image segmentation models was a deliberate choice aligned with the core objective of this research, which is to explore efficient solutions specifically tailored to the segmentation of infrared MG images, rather than to conduct a systematic comparison of all mainstream models. These models were chosen to systematically evaluate their performance in infrared MG image segmentation, leveraging their distinct architectural advantages, without modifications to their architectures. All models were trained using cross-entropy loss as the loss function and the Adam optimizer with a learning rate of 0.001 to ensure fairness and consistency in comparison results.

DeepLabV3 + is a deep convolutional neural network based on the encoder-decoder architecture. It ingeniously integrates atrous convolution and atrous spatial pyramid pooling (ASPP), effectively capturing multi-scale contextual information and thereby generating high-resolution segmentation masks (Chen et al., 2018a). As a classic encoder-decoder structure, U-Net retains multi-scale features through skip connections and is widely applied to various image segmentation tasks, especially demonstrating outstanding capabilities in the field of medical image segmentation (Ronneberger et al., 2015). U-Net++ is an improvement on U-Net. It introduces more complex skip connections and nested structures, significantly enhancing the fusion effect of features at different levels, making it perform particularly well in image segmentation tasks dealing with complex boundaries or fine structures (Zhou et al., 2018).

**Figure 2:**
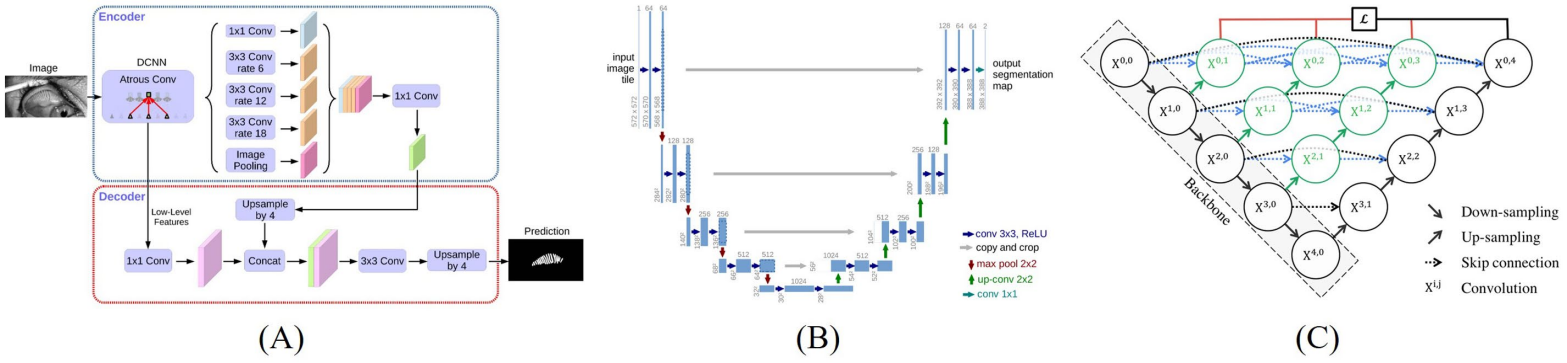
Architecture of three image segmentation algorithms, **(A)** DeepLabV3+ (Chen et al., 2018a), **(B)** U-Net (Ronneberger et al., 2015), **(C)** U-Net++ (Zhou et al., 2018).

Thereby, DeepLabV3 + outperformed in capturing global features and preserving contextual integrity, U-Net++ offered enhanced capability in detailing local features, and U-Net maintained stable results with relatively lower computational complexity. Figure 2 illustrates the structural differences and design philosophies among the three models.

### 3.3 Evaluate metrics

To evaluate the performance of the model on new data, metrics such as Precision, Intersection over Union (IoU), F1 score and Recall were used for the assessment on the test set, equations are shown in the Equations 1–4, where TP, TN, FP, and FN represent True Positives, True Negatives, False Positives, and False Negatives.


(1) $$\text{Precision}=\frac{\mathrm{TP}}{\mathrm{TP}+\mathrm{FP}}$$

(2) $$\mathrm{IoU}=\frac{\mathrm{TP}}{\mathrm{TP}+\mathrm{FP}+\mathrm{FN}}$$

(3) $$\begin{array}{c} F1\;\text{score}=\frac{2*\mathrm{TP}}{\left(2*\mathrm{TP}+\mathrm{FP}+\mathrm{FN}\right)} \end{array}$$

(4) $$\begin{array}{c} \text{Recall}=\frac{\mathrm{TP}}{\mathrm{TP}+\mathrm{FN}} \end{array}$$

### 3.4 Quantitative indicators measurement

We calculated the following indicators: gland width, gland length, gland distortion, gland number, gland area, density, loss ratio, nearest distance between adjacent glands, and degree of disorder as the morphological and distribution characteristics of the MG. The calculations were performed through image processing and contour analysis based on the gland images and the tarsus region images. Apply a morphological opening operation once using a 3 × 3 elliptical kernel to remove noise. Detect the contours and filter out those with an area of less than 10 pixels or with fewer than 4 points. Sort the contours from left to right according to the abscissa axis of their center of mass to ensure a consistent analysis order.

The number of glands directly reflects the remaining quantity of glands, and a decrease in number is a core feature of MGD. Glands are sorted from left to right by abscissa and marked one by one for quantitative counting.

**Figure 3:**
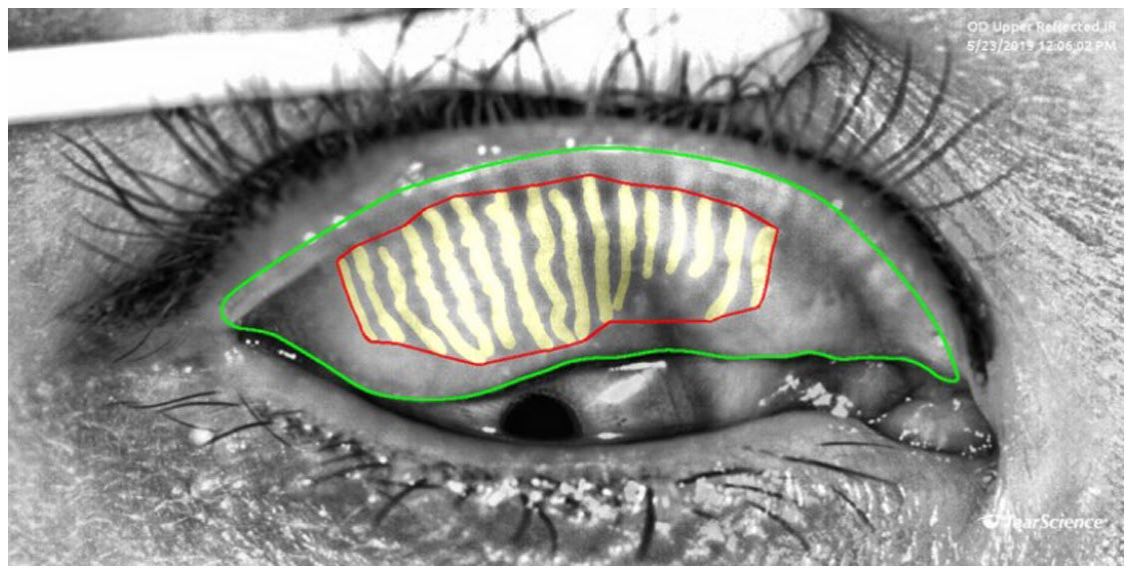
Examples of measurement of the area, density and loss ratio of the glands: the red area represents the gland area, gland density is calculated as the red area divided by the area enclosed by the green lines, The gland loss ratio is computed as the quotient of (the area bounded by the green contours minus the red contours) divided by the area bounded by the green contours.

Gland area represents the actual coverage of glands. A reduction in area indicates gland atrophy or loss, which is directly linked to decreased tear film stability. It is calculated by summing the areas of the outer boundaries of each gland, as shown by the yellow region in Figure 3. Gland density is calculated by dividing the total MG area by the tarsal region area, reflecting the abundance of glands per unit area. A decrease in density is a macroscopic manifestation of gland degeneration. Loss ratio refers to the proportion of missing gland area relative to the total tarsal area. It is calculated by subtracting the sum of all gland areas from the tarsal contour area and then dividing by the tarsal contour area. This index quantitatively evaluates the degree of gland loss by comparing the gland area with the entire tarsal area, serving as a core parameter for MGD diagnosis and treatment efficacy assessment.

**Figure 4:**
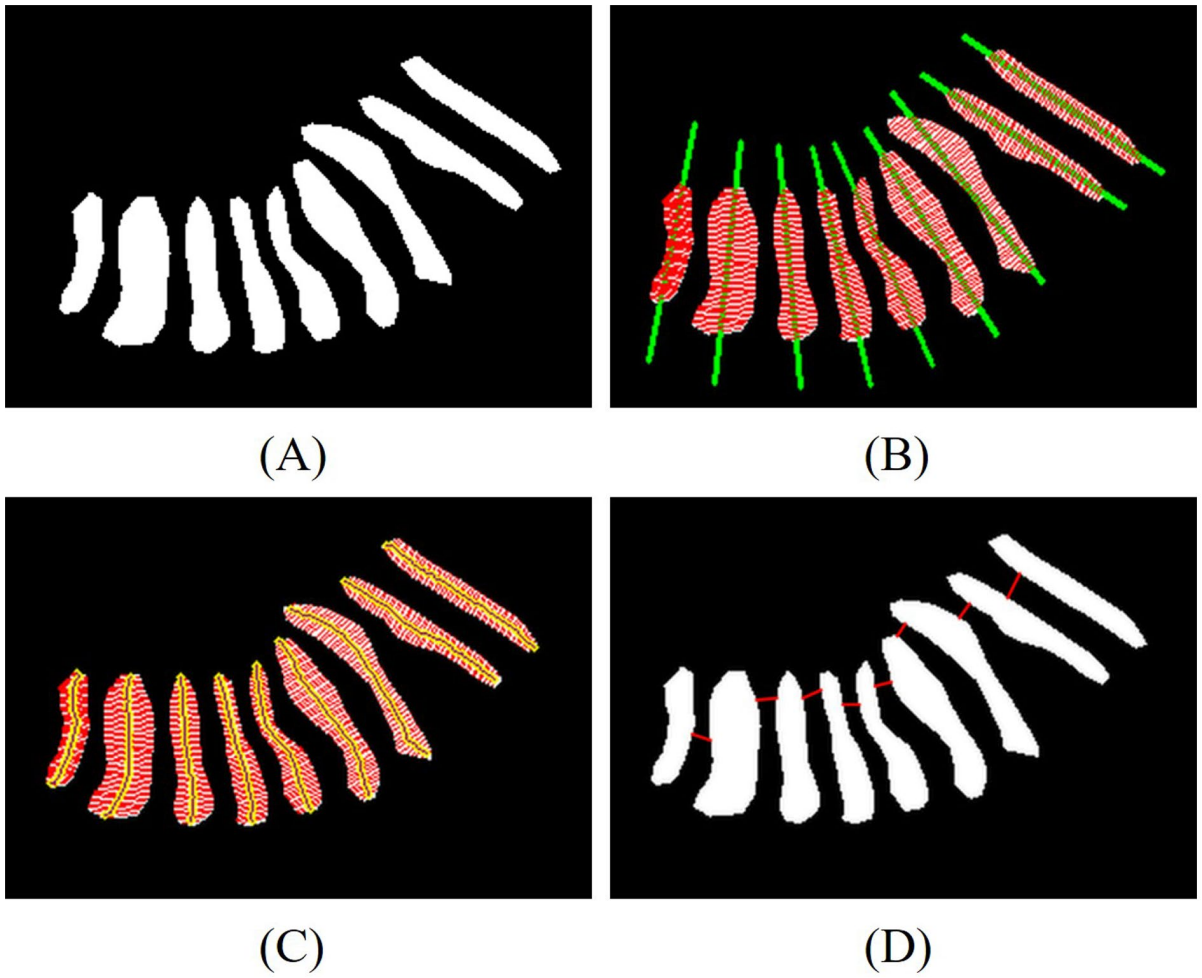
Examples of measurement of the length, width, distortion of the glands and the nearest distance between adjacent glands. **(A)** Eyelid annotated images, **(B)** line of principal direction and width, **(C)** line of width and length, **(D)** line of nearest distance between adjacent glands.

To calculate the length, width, and distortion of the gland, we have developed a novel computational framework that integrates Principal Component Analysis (PCA) with equidistant sampling, enabling systematic characterization of glandular morphology. As presented in Figure 4, this framework first extracts the gland’s principal direction via PCA and projects contour points to generate 50 equidistant sampling points. Gland width is computed as the average distance between intersection points of 50 perpendicular lines to the principal direction and the contour, while length is determined by summing Euclidean distances between midpoints of equidistant line segments. The distortion of the gland is represented by the discrete curvature of the length segments. We calculate the included angles based on three adjacent points and weight the distances of adjacent segments. The average curvature of all middle line points is taken as the distortion degree of the gland. Medically, gland width reflects changes in gland crosswise area, with reduced width commonly seen in gland atrophy or obstruction; gland length indicates the longitudinal extension of the gland, and shortened length may be associated with gland degeneration; gland distortion quantifies the regularity of gland morphology, and increased distortion indicates structural damage to the glands, which is positively correlated with the severity of MGD.

Another key innovation in our quantitative analysis lies in the development of two novel spatial distribution metrics is the nearest distance between adjacent glands and the degree of disorder, as illustrated in Figure 4D. After sorting the centroids of the glands, the Euclidean distance between the contour points of adjacent glands is calculated. The red lines indicate the nearest distance between adjacent glands, which help evaluate how glands are distributed in space. A larger distance between adjacent glands suggests that glands are more scattered, indirectly reflecting gland density. The degree of disorder is reflected by the standard deviation of the nearest distances between adjacent glands. A stronger disorder in distribution indicates a closer correlation with the gland damage pattern caused by MGD.

### 3.5 Statistical analysis

The Spearman’s correlation analysis method was adopted to explore the correlation between the indicators of MG and the MGD grade. By drawing the scatter plot of Spearman’s correlation, the strength of the association between each parameter indicator and the grade was analyzed. Among them, the correlation with a *p*-value less than 0.001 was statistically significant, indicating that these indicators may play an important role in the evaluation of MGD grade.

In addition, box plot visualization was used to analyze the distribution of parameter indicators in the classification of MG after segmentation, covering nine key indicators such as total gland area and gland loss ratio, to clearly present the data features across different grades. To better highlight the differences between groups at various grades for the parameter indicators extracted after segmentation, this method compared the data distributions of grades 0, 1, 2, and 3, revealing that changes in MG classification grades may be closely related to variations in parameter indicators. These variations were clearly shown through differences in the medians and interquartile ranges across the grades.

To evaluate the performance of the vs. of MG under different MGD grade, in this study, a logistic regression model was used, and the data was divided into a training set and a test set at a ratio of 8:2 to construct a multi-class classification model. By calculating ROC and AUC, the predictive performance of the indicators of MG under different grade was compared, and the robustness of performance evaluation was enhanced through 5-fold cross-validation. It shows that the indicators of MG have good predictive ability in distinguishing different grade, providing a reliable basis for the evaluation of MG function.

## 4 Results

### 4.1 Segmentation result

**Figure 5:**
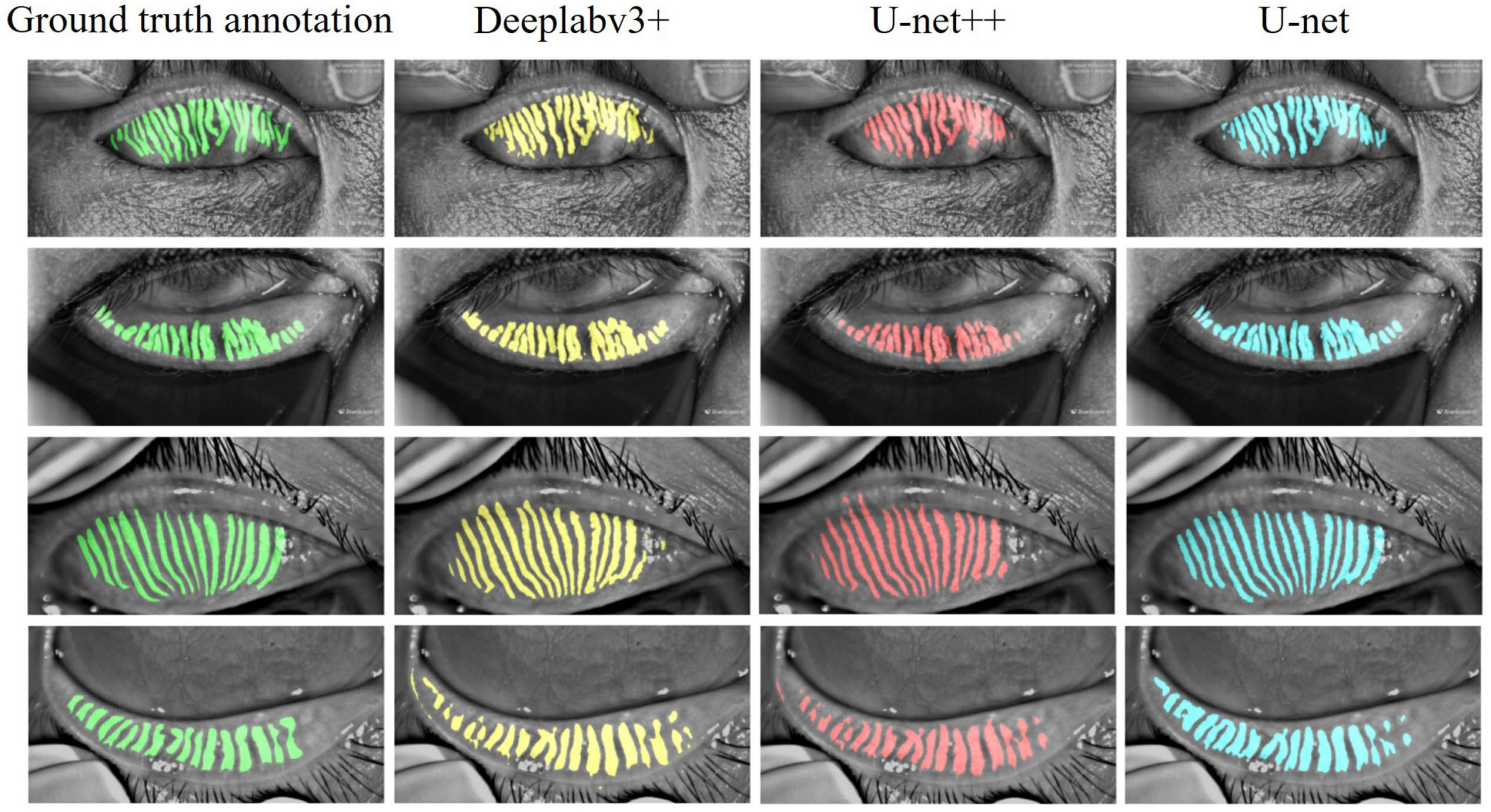
Case of model performance on images from the test set.

Three models were systematically evaluated the performance for MG segmentation tasks using test data. As shown in Figure 5, comparative cases between the segmentation results of these models after 100 epochs of training and manually annotated images on the test set are presented. The experimental results indicate that all three models can effectively identify MG structures, but significant differences exist in their ability to handle details. Specifically, U-Net demonstrates higher precision in segmenting complex gland edges and fine structures, particularly in the segmentation of lower eyelid images, where its results are closer to the manually annotated true value images. In contrast, DeepLabV3 + and U-Net++ exhibit less ideal segmentation performance when dealing with complex gland structures, with certain gaps in capturing edge details and tiny structures.

Table 1 summarizes the performance of three models DeepLabV3+, U-Net++, and U-Net in MG and eyelid segmentation tasks, with evaluation metrics including Precision, IoU, F1 score, and Recall. The experimental results show that U-Net demonstrates significant advantages in MG segmentation. Its IoU index reaches 0.72, higher than 0.70 for both DeepLabV3 + and U-Net++, indicating that U-Net has higher accuracy in handling overlapping segmentation regions. In terms of F1 score, U-Net also performs excellently at 0.82, while DeepLabV3 + and U-Net++ both achieve 0.81, suggesting that U-Net has better stability in balancing precision and recall.

**Table 1:** Performance of the MG segmentation method.

Metrics	DeepLabV3+		U-Net++		U-Net	
	Gland	Eyelid	Gland	Eyelid	Gland	Eyelid
Precision	0.82	0.96	0.83	0.96	0.84	0.97
Recall	0.83	0.97	0.84	0.96	0.85	0.97
IoU	0.70	0.93	0.70	0.93	0.72	0.94
F1 score	0.81	0.96	0.81	0.96	0.82	0.97

In contrast, according to Table 1, the three models exhibit minimal performance differences in eyelid segmentation, with IoU values ranging from 0.93 to 0.94. This indicates that eyelid region segmentation is relatively less challenging, with insignificant differences between models. These findings further validate that the complexity of MG structures significantly impacts model segmentation accuracy, while the relatively regular structure of the eyelid region enables all models to achieve satisfactory segmentation results.

### 4.2 Quantitative indicators and statistical analysis

**Figure 6:**
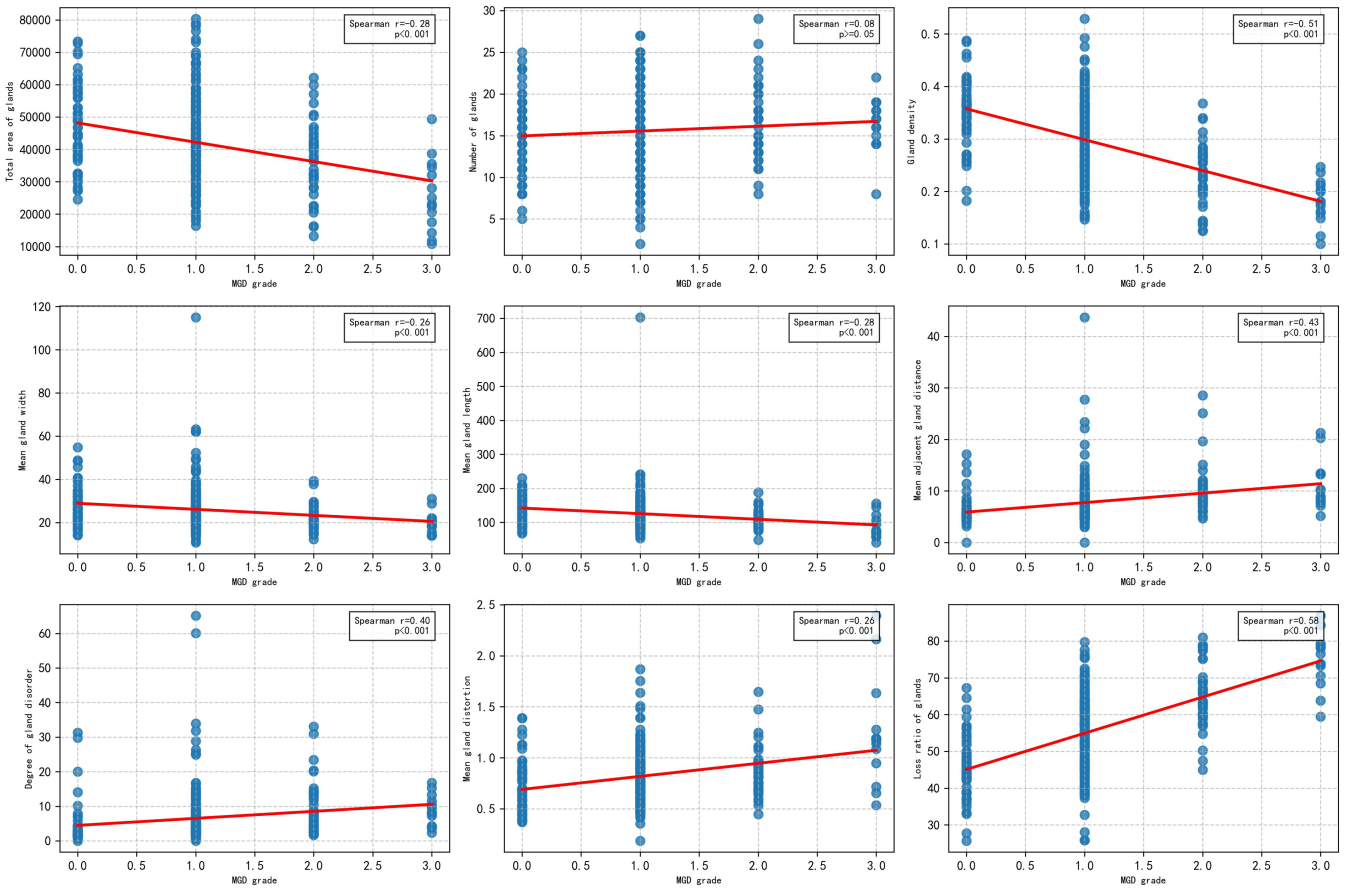
Scatter plots of Spearman’s correlation between MG indicators and MGD grade.

To explore the relationship between the morphological and distribution characteristics of MG and the severity of MGD, the Spearman correlation analysis was used to evaluate the correlation between the MG indicators obtained by algorithm segmentation outputs of the U-Net model with the best performance (IoU of 0.72 and F1 score of 0.82), and MGD grade. As shown in Figure 6, the figure display the scatter distributions of various quantitative indicators (including gland area, number, density, width, length, distance between adjacent glands, degree of disorder, distortion, and loss ratio) and MGD grade. The results showed that gland area, density, length, width, distortion, distance between adjacent glands, disorder degree, and loss ratio were significantly correlated with MGD grade, Spearman correlation coefficients ranged from 0.26 to 0.58 (*p* < 0.001), indicating that these indicators change significantly with the MGD severity and have strong correlations. In contrast, gland number (*r* = 0.08, *p* = 0.15) showed weak correlations with MGD grade, suggesting that may have limited diagnostic value in MGD grading.

**Figure 7:**
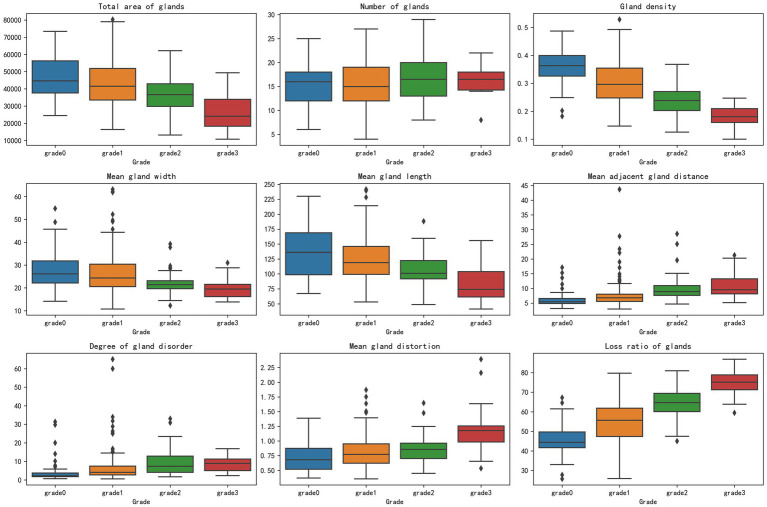
Box plots of parameter indicators across meibomian gland grades.

This study utilized box plot analysis to examine the distribution of nine parameter indicators in segmented MG images, revealing a clear gradient change across the grades. Specifically, indicators such as gland loss ratio and gland density demonstrated particularly prominent effects: the median gland loss ratio increased progressively from a lower value in the healthy group to a higher value in the severe group, with widening interquartile ranges and an increase in outliers, clearly reflecting the trend of gland degeneration due to worsening disease; conversely, gland density exhibited a decreasing pattern, with the median dropping from a higher value in the healthy group to a lower value in the severe group, and an expanded interquartile range indicating increased variability. The highly consistent distribution changes in these indicators provide strong evidence, supporting their potential for MGD in Figure 7.

Other indicators, such as total gland area and mean gland length, also showed a favorable gradient effect, with medians decreasing as the grade increased and interquartile ranges widening, highlighting morphological evidence of gland atrophy. Indicators like mean gland width, mean adjacent gland distance, mean gland distortion, and degree of gland disorder also displayed certain gradient changes, with medians showing a slight decreasing or increasing trend and interquartile ranges changing moderately, with fewer outliers, suggesting that while these indicators reflect some role in MGD progression, the effect is not highly pronounced. Only the gland number showed a relatively moderate distribution change, with minimal median fluctuation, no significant expansion of the interquartile range, and a relatively uniform distribution of outliers, possibly influenced by sample variability, indicating lower sensitivity in grade classification.

The box plots visually demonstrated inter group differences across grades, particularly the pronounced differences between the healthy group and the moderate to severe group, providing important morphological evidence for MGD detection. These findings reinforce the close relationship between MG classification grades and the variability of parameter indicators, especially between the healthy group and the moderate to severe group, where a significant reduction in total gland area, decreased gland density, narrowed mean gland width, shortened mean gland length, increased mean adjacent gland distance, elevated mean gland distortion, and a significant rise in gland loss ratio all reflect the worsening trend of disease severity. The difference in gland number remained consistently insignificant. In contrast, gland density and gland loss ratio emerged as promising potential markers for distinguishing healthy individuals from those with moderate to severe MGD.

**Figure 8:**
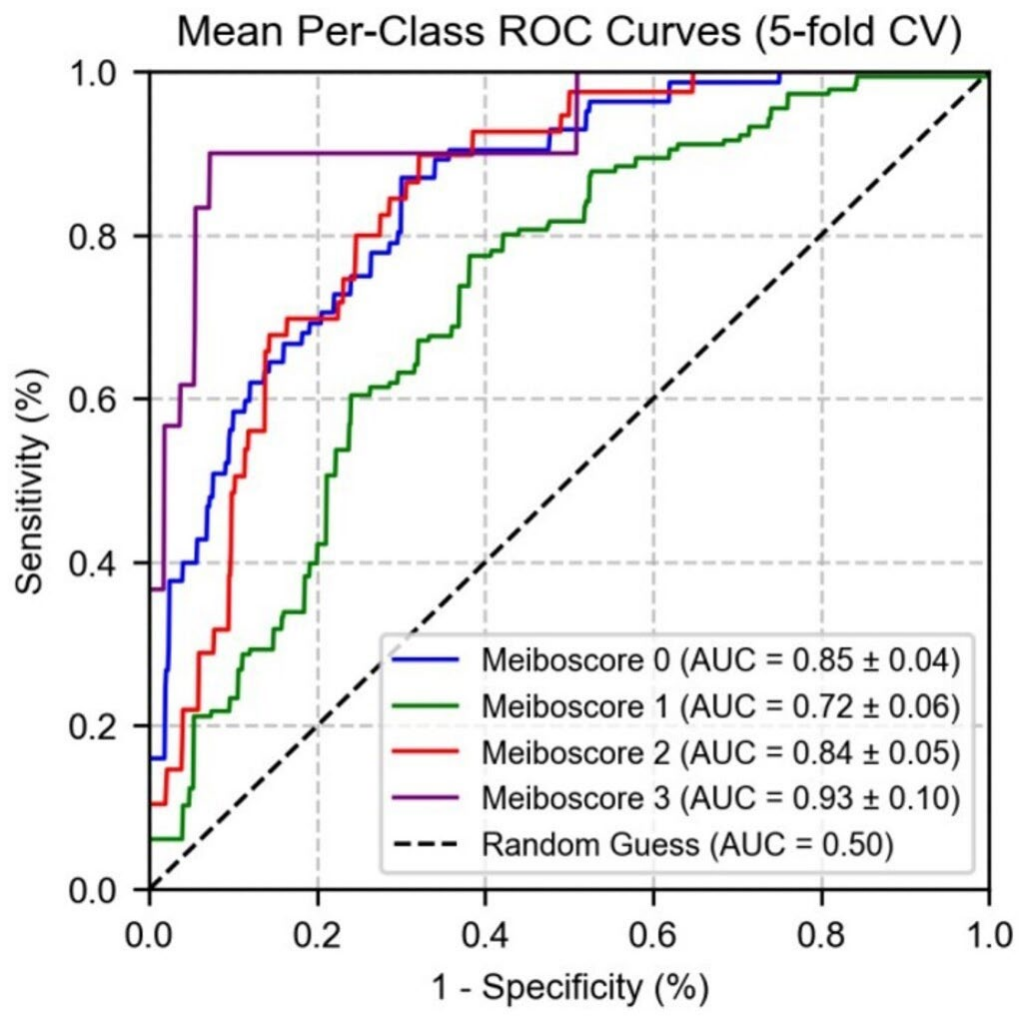
The ROC curve of the logistic regression for the diagnostic indicators of MGD grade.

Subsequently, a logistic regression model using MG indicators and MGD grade to further validate the predictive performance of these indicators across different grades. As shown in Figure 8, the area under the curve values of the indicators for grades 0, 1, 2, and 3 were 0.89 ± 0.02, 0.76 ± 0.03, 0.85 ± 0.02, and 0.94 ± 0.01, respectively. These results indicate that the indicators exhibit the strongest differential ability at higher severity levels (particularly grade 3), with the highest AUC for grade 3 MGD, demonstrating high sensitivity and specificity in diagnosing severe MGD.

**Figure 9:**
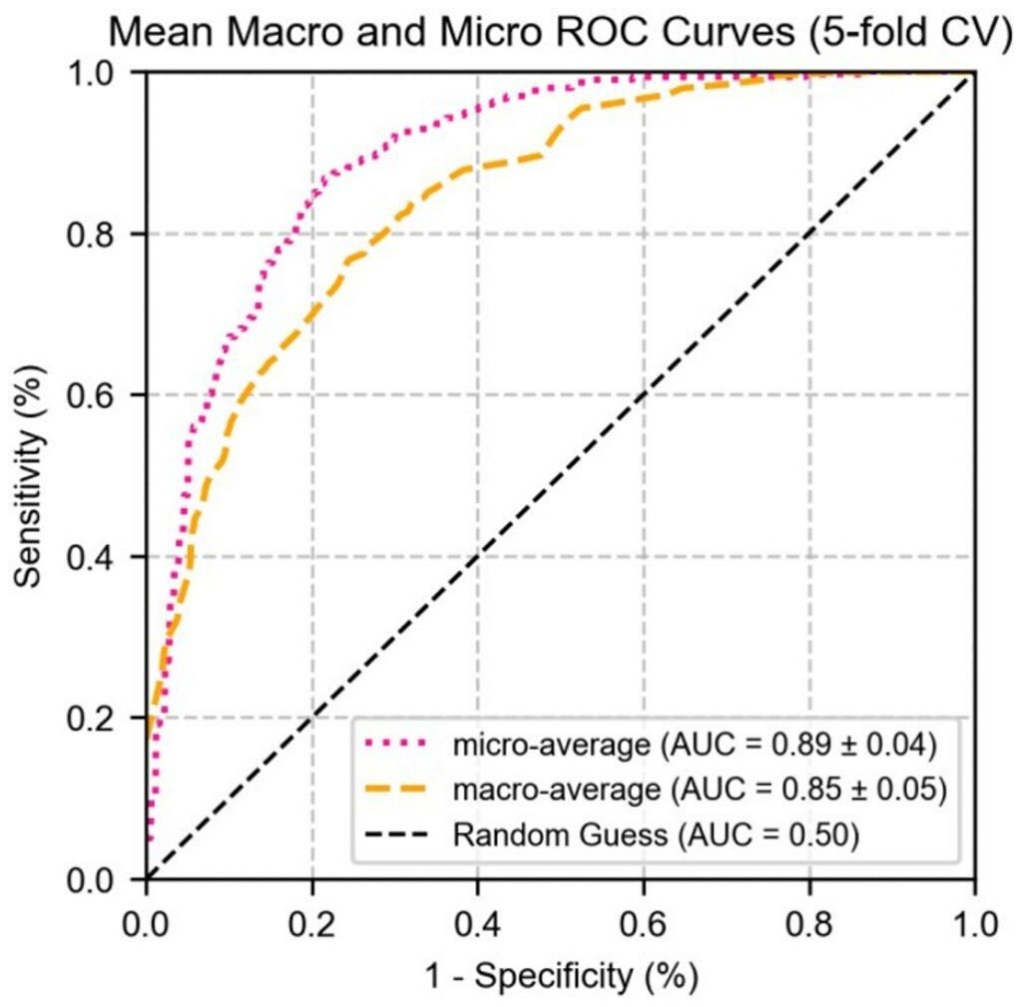
The ROC curve of logistic regression for micro/macro-average performance of test set parameter indicators.

Figure 9 shows the performance of micro-average (AUC = 0.87 ± 0.02) and macro-average (AUC = 0.86 ± 0.03), both of which significantly outperformed random guessing (AUC = 0.50). Combined with Figure 7, these results demonstrate that MG indicators exhibit excellent differential ability in multi-class prediction, particularly in distinguishing healthy individuals (grade 0) from moderate to severe MGD patients (grades 2–3). The robustness of the model was further validated through 5-fold cross-validation, providing a reliable quantitative basis for the automated diagnosis and grading of MGD.

## 5 Discussion

This study implements automated segmentation of MG infrared images based on deep learning algorithms, and carries out quantitative analysis on binary segmented images, providing an innovative solution for the diagnosis and grading of MGD. The experimental data of this study were collected by the Lipi View II ocular surface interferometer and the Oculus Keratograph 5 M (Oculus GmbH, Wetzlar, Germany). The technical characteristic differences between the two devices form the heterogeneity basis of the datasets, providing key support for verifying the adaptability of the algorithm under different imaging mechanisms. Experimental data show that compared with the DeepLabV3 + and U-Net++ models, U-Net demonstrates higher accuracy in MG image segmentation tasks, especially showing unique advantages in processing irregular gland structures. Advanced preprocessing techniques were not employed to maintain computational simplicity and experimental reproducibility.

The quantitative indicators derived from the segmentation results serve as critical metrics for the objective assessment of MGD. Parameters such as gland area, density, width, length, distortion, inter-gland distance, disorder degree, and gland loss ratio exhibited significant variation across different MGD severity levels, effectively capturing the pathological features associated with disease progression. Notably, the total number of glands did not demonstrate a significant correlation with MGD severity. This may be attributed to the fact that gland count remains relatively stable during the early or mild stages of MGD, whereas morphological and spatial distributional changes are more sensitive markers that better reflect the dynamic and progressive nature of the disorder.

The high differential ability of the logistic regression model further identifies the application value of the quantitative indices extracted from MG infrared image segmentation in the grading of MGD. The model demonstrated the strongest predictive efficacy in severe MGD (grade 3) cases, which may be attributed to the significant abnormalities in MG morphology and distribution in severe patients, enabling quantitative indices to more clearly distinguish between pathological and healthy states. In contrast, the model showed slightly lower predictive ability in mild MGD (grade 1) cases, likely due to the subtle glandular changes in mild MGD, which increase the diagnostic complexity based on quantitative indices.

In previous studies on quantitative indices of the MG, most have focused on traditional parameters such as gland count, length, width, and area, while some have involved relatively novel indices like distortion and density. The innovations and advantages of the index system in this study are mainly reflected in three aspects: first, it improves the calculation method of traditional morphological indices by creatively proposing a PCA approach to calculate gland length, width, and distortion. Compared with traditional manual measurement or simple geometric fitting, this method can more accurately capture the natural extension direction and morphological characteristics of glands, and particularly describe irregular gland structures such as curved or branched ones in a way that better conforms to real pathological morphology, reducing measurement deviations caused by morphological complexity; second, it fills the gap in quantifying spatial distribution characteristics. By introducing indices of adjacent gland distance and disorder degree, it realizes quantitative analysis of the spatial distribution characteristics of glands for the first time. Traditional studies have mostly focused on the morphology of individual glands, but this study found that the spatial arrangement patterns of glands are closely related to the progression of MGD. For example, patients with severe MGD often show clustered atrophy of glands, with significantly increased adjacent distances and disorder degrees. These characteristics, which cannot be reflected by simple morphological indices, provide a new diagnostic dimension for disease grading; third, it proposes a comprehensive pathological index. The newly constructed gland loss ratio, by quantifying the proportion of areas where glands have disappeared in the total gland distribution area, can simultaneously reflect multiple pathological changes such as gland atrophy and sparse distribution. It overcomes the limitation that traditional single indices can only describe local features, and more comprehensively reflects the overall degradation trend of glands during the course of MGD.

While this study concentrated on the morphological and distributional characteristics of MG derived from image segmentation, it overlooked two critical assessment components. It did not integrate functional indicators essential for a comprehensive evaluation of MGD, such as gland secretion quality and expressible secretion ability, which could enhance diagnostic accuracy. Additionally, the assessment excluded MG orifice obstruction, a clinically significant feature in MGD pathophysiology. This omission arises from two key limitations: (1) infrared imaging technology and the current segmentation algorithms are optimized for structural analysis, making it challenging to detect functional or dynamic features like orifice obstruction, and (2) the study’s focus on structural metrics precluded the development of a holistic assessment system combining structural and functional insights. To address these gaps, future research will explore the integration of multi-modal imaging technologies or functional metrics, incorporating MG orifice obstruction as well as gland secretion quality and expressible secretion ability, to refine the assessment framework and improve the comprehensiveness of MGD severity evaluation, thereby strengthening support for early detection.

To address these gaps, future research will explore the integration of multi-modal imaging technologies and functional metrics, incorporating indicators such as MG orifice obstruction, gland secretion quality, and expressible secretion ability, to refine the assessment framework and enhance the comprehensiveness of MGD severity evaluation, thereby providing stronger support for early detection. Specific pathways to achieve this include, on one hand, expanding datasets to include multi-modal sources, such as combining infrared meibography with dynamic imaging modalities, to capture functional data like meibum flow dynamics and orifice patency without invasive procedures. On the other hand, developing advanced algorithms to build on existing image segmentation frameworks, enabling simultaneous extraction of structural features and functional indicators.

Second, the combination of public MGD-1 K datasets and internal datasets enhances data diversity by accounting for real-world variations in imaging devices. However, the relatively small scale of the internal datasets may limit the reliability and generalizability of the findings, particularly in cases of imbalanced MGD grading distributions or diverse patient population characteristics. Expanding the internal datasets in future studies would enable further validation of model performance across varied clinical settings and reduce the risk of overfitting associated with heavy reliance on public benchmark data. To improve model adaptability across diverse infrared imaging sources, future work will explore domain adaptation techniques to align feature distributions from different devices and expand data augmentation to simulate device specific imaging variations. Integrating larger multisource infrared datasets will further enhance robustness and generalizability.

Third, the segmentation accuracy of classical image segmentation models for gland structures remains suboptimal, especially for low-quality images, where challenges in accurately capturing gland edges can lead to errors in calculating certain indices. Future efforts will involve the adoption of more advanced models to improve segmentation precision, meeting the demands of more accurate diagnostic applications.

## 6 Conclusion

This study explored a deep learning-based diagnostic and grading method for MG quantitative indicators, comparing the performance of DeepLabV3+, U-Net, and U-Net++ models in processing infrared MG images. Among them, U-Net demonstrated the best performance when evaluated based on segmentation accuracy, achieving an IoU of 0.72 and an F1 score of 0.82 for MG segmentation, particularly excelling at capturing complex gland edges and fine structures. Quantitative indicators extracted from segmentation results were significantly correlated with MGD grade (Spearman correlation coefficients ranging from 0.26 to 0.58, *p* < 0.001), indicating a close association with the severity of MGD. Box plot analysis intuitively revealed the clear gradient distribution changes of these indicators across different MGD grades, and this variation can be clearly reflected through the median separation degree, interquartile range overlap status, and outlier distribution characteristics of the parameters in each group, highlighting their diagnostic value. The logistic regression model showed excellent predictive performance, with AUC values of 0.89 ± 0.02, 0.76 ± 0.03, 0.85 ± 0.02, and 0.94 ± 0.01 for grades 0, 1, 2, and 3, respectively. Micro-average and macro-average AUC reached 0.87 ± 0.02 and 0.86 ± 0.03, with model robustness confirmed via 5-fold cross-validation. These results demonstrate that the method significantly enhances the objectivity, efficiency, and reproducibility of MGD diagnosis and grading. The MG quantitative indicator method proposed in this study not only advances ophthalmic diagnostics but also lays a solid technical foundation for broader applications in the medical imaging field.

## Data Availability

The original contributions presented in the study are included in the article/supplementary material, further inquiries can be directed to the corresponding authors.
